# Taxane & cyclophosphamide vs anthracycline & taxane-based chemotherapy as adjuvant treatment for breast cancer: a pooled analysis of randomized controlled trials by the Hellenic Academy of Oncology

**DOI:** 10.18632/oncotarget.26632

**Published:** 2019-02-05

**Authors:** Panagiotis Ntellas, Nikolaos Spathas, Sofia Agelaki, Elias Zintzaras, Emmanouil Saloustros

**Affiliations:** ^1^ Department of Medical Oncology, University Hospital of Ioannina, Ioannina, Greece; ^2^ Department of Biostatistics and Clinical Bioinformatics, Faculty of Medicine, University of Thessaly, Larissa, Greece; ^3^ 2nd Department of Medical Oncology, University Hospital Attikon, Athens, Greece; ^4^ Department of Medical Oncology, University General Hospital of Heraklion, Heraklion, Crete, Greece; ^5^ Department of Oncology, University General Hospital of Larissa, Larissa, Greece

**Keywords:** early breast cancer, adjuvant, anthracycline, taxane, non-Inferiority

## Abstract

**Background:**

Adjuvant chemotherapy has an indisputable value for early breast cancer patients. Anthracycline and taxane-based regimens (TaxAC) have not been proven superior to taxane & cyclophosphamide (TC), a less toxic combination. Our objective was to estimate the cumulative evidence for non-inferiority of TC against TaxAC, in the adjuvant setting of patients with HER2-negative, breast cancer.

**Results:**

Overall, 7,341 patients were included in this analysis. Superiority of TaxAC or non-inferiority of TC was not established either for the overall population (DFS HR, 1.11; 95% CI, 0.95–1.30; *p* = 0.18), or for the node-negative patients (HR, 1.05; 95% CI, 0.82–1.34; *p* = 0.71). A difference in DFS of 1.28% (TC DFS, 89.04%; 95% CI, 88%–90% & TaxAC DFS, 90.32%; 95% CI, 89%–91%) was found in favor of TaxAC. Lower risk of death was not established for either treatment regimen (OS-HR, 1.02; 95% CI, 0.82–1.25; *p* = 0.88). Overall, the toxicity profile favored TC.

**Conclusion:**

Although non-inferiority of TC was not proven, superiority of TaxAC is still questioned. The present analysis narrows the risk of recurrence between the treatment groups. Considering TC has a more favorable safety profile, the question as to which treatment regimen should be preferred under what circumstances, needs to be individualized according to patients’ characteristics and desires.

**Methods:**

Treatment efficacy data from The ABC trials, the Plan B trial and a trial by the Hellenic Oncology Research group (HORG) were pooled. Disease free survival (DFS) and overall survival (OS) were scrutinized. A HR of 1.18 for TC versus TaxAC was chosen to demonstrate inferiority.

## INTRODUCTION

Breast cancer is the most common malignant tumor and the second cause of cancer related death in women [1–3]. Early breast cancer (EBC) [4] defined as the absence of malignant spread beyond the breast and the axillary lymph nodes is the stage of the disease most frequently diagnosed [1]. The implementation of adjuvant therapy based on combinations of cytotoxic drugs, endocrine and biologic therapies for the eradication of microscopic systemic disease, has a major impact on recurrence, disease-free survival (DFS) and overall survival (OS) of women with EBC [5–10].

The type of adjuvant therapy is based upon both tumor (i.e. histology, stage, HER2 & endocrine receptors status, recurrence risk score) and patient characteristics (i.e. age, menopausal status, comorbidities). Before deciding for the administration of adjuvant chemotherapy physicians balance the individual risk of recurrence and the relative risk reduction, with the toxicity of the treatment. [8, 11].

Anthracycline-based combinations have been used in the treatment of early breast cancer for more than three decades [8] and are generally more effective than earlier combinations like the cyclophosphamide-methotrexate-fluorouracil (CMF) regimen [9, 12]. Along with anthracyclines, taxanes have emerged as particularly active cytotoxics against breast cancer [13]. Adjuvant polychemotherapy with taxanes given concurrently or sequentially with anthracyclines has resulted in reduced recurrence and mortality rates compared to anthracycline-based regiments alone [6, 8, 10]. Consequently, taxane-anthracycline combinations are now widely used as adjuvant chemotherapy both for Her2-negative and Her2-positive early breast cancer [6, 8, 14].

Nevertheless, safety concerns regarding cardiotoxicity and secondary malignancies linked to anthracycline use, along with promising data from taxane-based, non-anthracyclines containing regimens have called the role of anthracyclines into doubt [8]. A less toxic non-anthracycline regimen is very appealing both for physicians and patients, especially those with reduced physiological reserves and significant comorbidities such as the elderly population [13]. The docetaxel-cyclophosphamide (TC) combination is a non-anthracycline containing regimen that has demonstrated superior efficacy in terms of DFS and OS compared to doxorubicin-cyclophosphamide (AC) [15–17]. However, AC is considered obsolete with contemporary standards [5] and AC in sequence or concurrent with a taxane (TaxAC) is usually preferred to treat high risk patients [8, 15, 18–20].

To date, three major studies have addressed the efficacy of TC compared to TaxAC for early stage HER2-negative breast cancer; namely the ABC trials, the WSG Plan B trial and the Hellenic Oncology Research Group (HORG) trial, demonstrating mixed results [5, 21, 22]. In such a situation, a pooled analysis of the available data may help resolve controversial issues given its capacity to provide more accurate, usually with narrower confidence interval (CI), estimates of the treatment effect and could also identify the causes of heterogeneity among different trials [6]. In this study we aimed to address the cumulative evidence for non-inferiority of the anthracycline-free regimen TC against TaxAC in the adjuvant setting of patients with HER2-negative, invasive breast cancer.

## RESULTS

### Study characteristics and patients’ demographics

The efficacy outcomes of the ABC trials and the HORG trial were published in 2017 and 2016 respectively, whereas the WSG Plan B trial was presented in the American Society of Clinical Oncology (ASCO) annual meeting in 2017. All of the included trials had a matching experimental arm that consisted of docetaxel (75 mg/m^2^) and cyclophosphamide (600 mg/m^2^) for 6 cycles (TC). In the HORG trial, the comparison arm consisted of epirubicin (75 mg/m^2^), 5-fluorouracil (500 mg/m^2^) and cyclophosphamide (500 mg/m^2^) for four cycles, followed by four cycles of docetaxel (75 mg/ m^2^). In the WSG Plan B trial, the comparison arm consisted of epirubicin (90 mg/m^2^) and cyclophosphamide (500 mg/m^2^) for four cycles, followed by 4 cycles of docetaxel (100 mg/m^2^). Among the ABC trials, in the USOR 03-090 trial, the anthracycline arm consisted of docetaxel (75 mg/m^2^), doxorubicin (50 mg/m^2^) and cyclophosphamide (500 mg/m^2^) for 6 cycles (TAC); in the B-46-I/USOR 07132 trial, patients were randomized to receive TC, TAC or TC plus Bevacizumab, but only data concerning the comparison of TC to TAC were analyzed; finally, in the NSABP B-49 trial patients in the anthracycline arm were given a choice between TAC for 6 cycles, or AC every 3 weeks for four cycles followed by paclitaxel (80 mg/m^2^) weekly for 12 doses, or AC every 2 weeks for four cycles followed by paclitaxel (80 mg/m^2^) weekly for 12 doses, or AC every 2 weeks for four cycles followed by paclitaxel (175 mg/m^2^) every 2 weeks for four cycles. Median follow-up was 3.3 years for the ABC trials, 3.9 years for the HORG trial, and 5 years for the WSG-Plan B trial.

**Figure 1:**
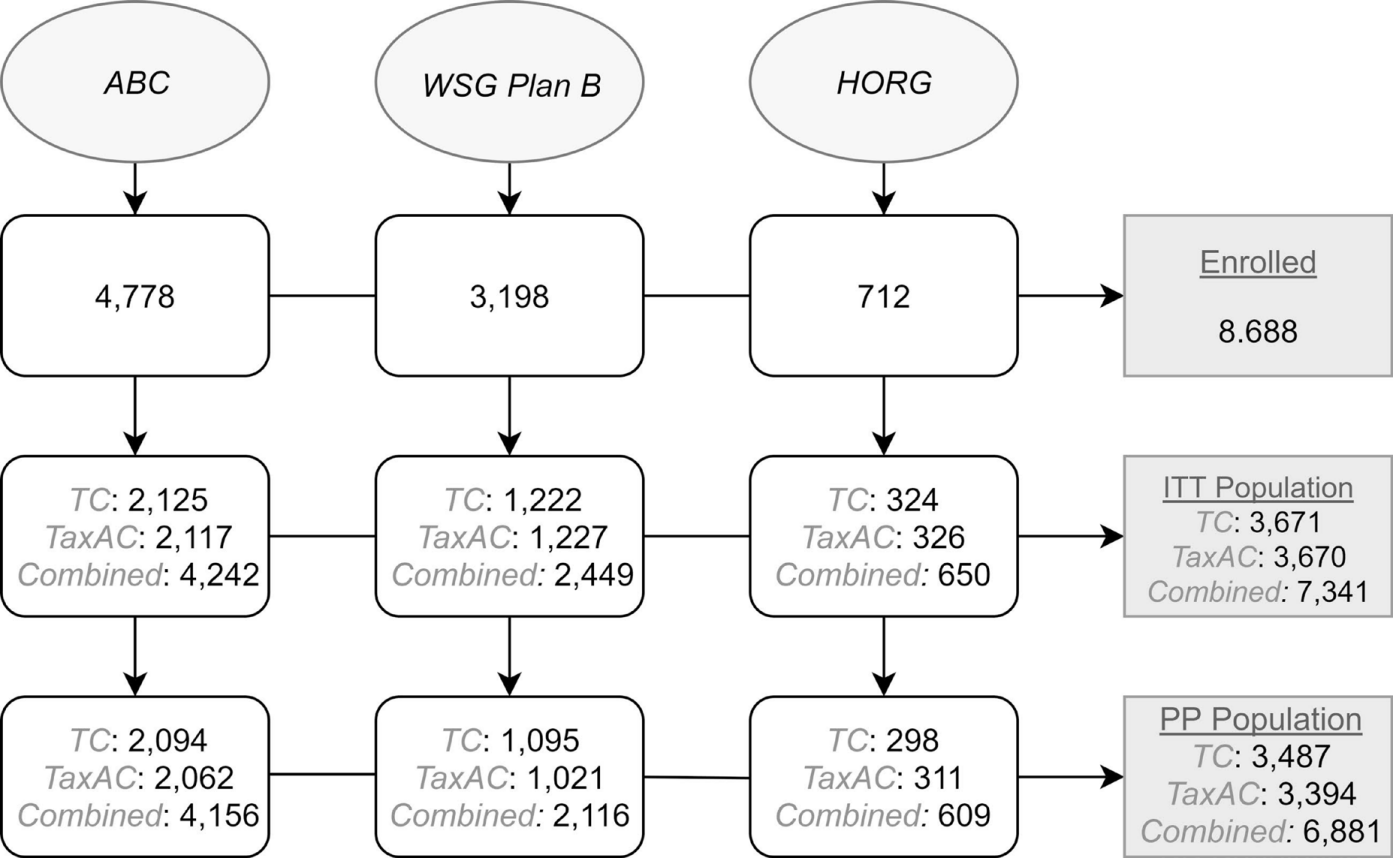
Flow diagram of included studies ITT = intention to treat, PP = per protocol, TC = taxane & cyclophosphamide, TaxAC = taxane & cyclophosphamide & anthracycline.

A total of 8.688 patients were collectively enrolled in the trials. Overall, 7.341 patients, that composed the intention to treat (ITT) population, were available for the pooled analysis; ultimately, only 6.881 patients completed the intended treatment (see Figure 1. Flowchart). Patients characteristics and survival are illustrated in Table 1. Breast conserving surgery was the preferred method of surgical treatment. Cumulatively, 3,660 patients had grade I/II tumors and 3,390 patients had grade III. Estrogen receptors were positive in tumors from 5,444 patients, while only 1,807 patients had ER-negative tumors. 3,144 patients had no infiltrated lymph nodes; 1 to 3 positive lymph nodes were detected in 3,074 patients; 4 to 9 positive lymph nodes were detected in 812 patients and only 264 patients had more than 10. Throughout, most breast cancer patients had up to three lymph nodes infiltrated. Also, 63% of the included patients were post-menopause.

**Table 1:** Patient & survival characteristics

Studies	Median follow-up	Grade *(n)*		Endocrine receptor *(n)*		Lymph nodes *(n)*				Menopausal status *(n)*		Surgery *(n)*		DFS rates *(%)*	
	*(in years)*	*I/II*	*III*	*Negative*	*Positive*	*N0*	*N1*	*N2*	*N3*	*Pre-*	*Post-*	*BCS*	*Mastectomy*	*TC*	*TaxAC*
*Plan B*	5	1323	1034	445	2004	1441	832	135	41	868	1388	1985	459	90	90
*ABC*	3.3	1954	2120	1288	2868	1686	1836	478	165	n/a	n/a	n/a	n/a	88.2	90.7
*HORG*	3.9	383	236	74	572	n/a	414	179	57	199	451	326	324	91.1	89.5
*Overall*	n/a	3660	3390	1807	5444	3144	3074	812	264	1067	1839	2311	783	89.04	90.32

N0 = zero lymph-nodes, N1 = 1 to 3 lymph-nodes, N2 = 4 to 9 lymph-nodes, N3 = 10 or more lymph-nodes.

Abbreviations: BCS = breast conserving surgery, DFS = disease free survival, TC = taxane & cyclophosphamide, TaxAC = taxane & cyclophosphamide & anthracycline.

### Primary endpoint (disease free survival)

**Figure 2:**
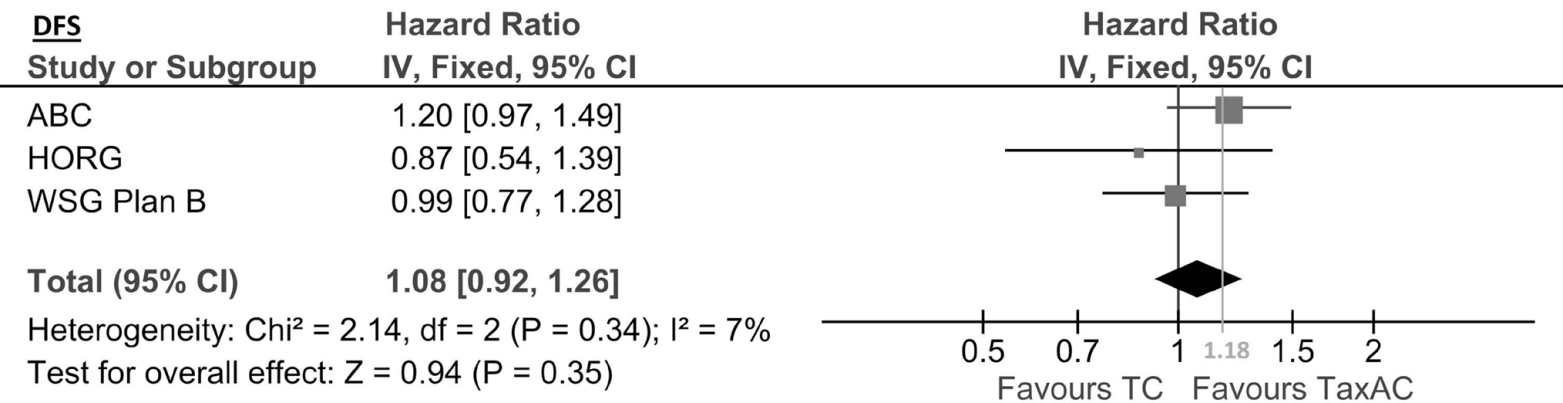
Disease free survival DFS = disease free survival, CI = confidence interval, 1.18 = non-inferiority margin, TC = taxane & cyclophosphamide, TaxAC = taxane & cyclophosphamide & anthracycline.

Primary focus of this analysis was to determine if the anthracycline-free regimen TC is non-inferior to TaxAC with regards to DFS. By combining the available ITT data (7,341 patients) we found a pooled-fixed effect HR of 1.08 (95% CI, 0.92–1.26, *p* = 0.35). The result did not demonstrate a statistically significant difference of the treatment effects of TC and TaxAC on DFS, meaning that no treatment regimen was found to be superior to the other. However, the upper limit of the 95% CI from the pooled analysis exceeded the non-inferiority threshold of 1.18, thus non-inferiority of TC could not be proven (Figure 2. Disease Free Survival). Synthesis of the available data for the rate of survival at the 5th, 4th and 3rd year for the Plan B, ABC and HORG trials respectively produced a pooled DFS rate of 89.04% (95% CI, 88% - 90%) for TC and 90.32% (95% CI, 89%–91%) for TaxAC, indicating an absolute difference in survival of 1.28% in favor of TaxAC. Fixed effects was the selected model as indicated by the absence of heterogeneity (*Q*-test *p* = 0.34, I^2^ = 7%), which was as expected considering the relative consistency of the patients characteristics between the RCTs. No statistically significant publication bias was detected by Egger’s test (*p* = 0.9), while visual inspection of the funnel plot revealed no evidence of asymmetry (Supplementary Figure 1. Publication Bias).

### Secondary endpoints (overall survival, node-negative patients)

The available survival data from the 7,341 patients were combined to determine the treatment effect on overall survival; pooled analysis yielded a fixed-effects HR of 1.02 (95% CI, 0.82–1.25, *p* = 0.88). At the time point that this analysis was carried out, no statistically significant difference was found between the treatment regimens in terms of overall survival (Supplementary Figure 2. Overall Survival & Node-Negative DFS). Between study heterogeneity was not observed (*Q*-test *p* = 0.80, I^2^ = 0%).

Out of the 3 studies only ABC trials and Plan B trial included patients without infiltrated lymph nodes. Overall, the available survival data from 3,127 patients were included in the subgroup pooled-analysis of the treatment effect on DFS for the lymph node negative population and provided a fixed-effects HR for DFS of 1.05 (95% CI, 0.82–1.34; *p* = 0.77); The difference found on DFS for the node-negative population was not statistically significant, however, non-inferiority of TC could not be proven (Supplementary Figure 2. Overall Survival & Node-Negative DFS). No heterogeneity was observed between the studies (*Q*-test *p* = 0.88, I^2^ = 0%).

### Toxicity

Pooled toxicity analysis from all 5 RCTs is listed in Table 2. Neutropenia, mostly grade 3–4, was common in both groups. Anemia was frequently observed but didn’t seem to pose much of a problem since mainly grade 1–2 events were reported, with a higher rate, in the TaxAC group (OR = 1.3, *p* = 0.001). Thrombocytopenia was rarely observed (TaxAC, 1.84%; TC, 0.29%), but was higher in the TaxAC group (grade 1–2: OR = 1.8, *p* = 0.001, grade 3–4: OR = 6.4, *p* < 0.001). Leukopenia, grade 3–4 events were mostly observed in the TaxAC group (OR = 1.2, *p* = 0.002), whereas febrile neutropenia grade 3–4 events were more common in the TC group (OR = 0.74, *p* = 0.005). Grade 3–4 immune-related or hypersensitivity reactions such as allergy (OR = 0.35, *p* < 0.001), rash (OR = 0.4, *p* = 0.002) and skin toxicity (OR = 0.2, *p* < 0.02) were mainly associated with TC. Nausea (grade 1–2: OR = 1.7, *p* < 0.001; grade 3–4: OR = 2.6, *p* < 0.001), vomiting (grade 1–2: OR = 2.5, *p* < 0.001, grade 3–4: OR = 2.2, *p* < 0.001), diarrhea (grade 1–2: OR = 1.5, *p* < 0.001), mucositis (grade 1–2: OR = 1.6, *p* = 0.003; grade 3–4: OR = 2.8, *p* < 0.001), hand/foot syndrome (grade 3–4: OR = 1.9, *p* = 0.01), fatigue (grade 1–2: OR = 1.2, *p* = 0.02; grade 3–4: OR = 1.7, *p* < 0.001), neurotoxicity (grade 3–4: OR = 1.8, p=0.001), infection (grade 1–2: OR = 1.7, *p* = 0.006), pain (grade 3–4: OR = 1.3, *p* = 0.04) and cardiotoxicity (grade 3–4: OR = 2.28, *p* = 0.01) were observed at a higher rate in the TaxAC arm. Data concerning development of secondary malignancies have been so far reported only for ABC trials, and as expected implicate the use of anthracycline containing regimens with the development of secondary hematologic malignancies.

**Table 2:** Toxicity profile

	Grade 1-2				Grade 3-4			
	*TaxAC %*	*TC %*	*OR*	*p*	*TaxAC %*	*TC %*	*OR*	*p*
**Neutropenia**	5.69	5.02		*0.41*	28.24	28.98		*0.48*
**Anemia**	28.56	23.42	***1.3***	***0.001***	1.34	0.87		*0.12*
**Thrombocytopenia**	5.63	3.14	***1.8***	***0.001***	1.84	0.29	***6.4***	***<0.001***
**Leucopenia**	1.82	2.73		*0.14*	24.31	21.12	***1.2***	***0.002***
**Febrile Neutropenia**	0.06	0.20		*0.32*	4.64	5.84	***0.74***	***0.005***
**Nausea**	21.77	14.25	***1.7***	***<0.001***	3.10	1.19	***2.6***	***<0.001***
**Vomiting**	10.37	4.41	***2.5***	***<0.001***	1.96	0.89	***2.2***	***<0.001***
**Diarrhea**	13.22	9.03	***1.5***	***<0.001***	2.87	2.78		*0.8*
**Mucositis**	7.32	5.02	***1.6***	***0.003***	2.44	0.89	***2.8***	***<0.001***
**Constipation**	4.20	5.28		*0.16*	0.33	0.13		*0.25*
**Allergy**	3.45	4.75		*0.07*	0.67	1.90	***0.35***	***<0.001***
**Rash**	3.73	10.44	***0.33***	***<0.001***	0.67	1.61	***0.4***	***0.002***
**Skin toxicity**	2.64	3.88	*0.67*	*0.058*	0.13	0.66	***0.2***	***0.02***
**Hand/Foot syndrome**	0.67	1.33		*0.7*	1.23	0.66	***1.9***	***0.013***
**Nail toxicity**	3.12	3.94		*0.2*	0.06	0.13		*0.5*
**Conjuctivitis**	0.81	0.87		*0.86*	0	0	*n/a*	*n/a*
**Edema**	3.93	4.55		*0.4*	0.27	0.33		*0.7*
**Fatigue**	27.61	24.02	***1.2***	***0.026***	5.26	3.17	***1.7***	***<0.001***
**Neurotoxicity**	5.08	5.08		*0.99*	2.67	1.53	***1.8***	***0.001***
**Infection**	5.92	3.50	***1.7***	***0.006***	4.27	4.22		*0.9*
**Arthralgia/myalgia**	10.27	16.23	***0.59***	***<0.001***	2.85	2.47		*0.3*
**Pain**	11.75	12.56		*0.5*	3.96	3.03	***1.3***	***0.04***
**Cardiotoxicity**	0.88	1.07		*0.6*	0.75	0.33	***2.28***	***0.015***

Abbreviations: OR = odds ratio, TC = taxane & cyclophosphamide, TaxAC = taxane & cyclophosphamide & anthracycline.

## DISCUSSION

To date several publications have addressed the appropriate adjuvant treatment for patients with EBC [6–10, 14]. Along with anthracyclines, taxanes are the most effective agents [6, 8, 13, 15]. The incorporation of taxanes to anthracycline containing regimens seems to provide superior treatment benefits when compared to many other chemotherapy combinations, while superiority of TaxAC is not evident against TC [6]. Furthermore, concerns have been raised regarding the toxicity of triple combination regimens, implicating anthracycline use with cardiac toxicity and secondary hematological malignancies [5, 10, 19].

The ABC trials [21], the HORG trial [5] and the WSG Plan B [22] trial have all set out to compare the efficacy of the TC regimen against TaxAC in the adjuvant setting of HER2-negative EBC. The anthracycline containing regimens were not found to be superior to TC, while non-inferiority of TC was ambiguous in these individual studies. Therefore, our study by combining the available data from these RCTs, serves as the next logical step in the evaluation process of TC. In our pooled-analysis the risk of recurrence and the risk of death seemed to favor TaxAC, without, however, providing statistical significance, meaning that no treatment arm was found to be superior to the other in terms of DFS or OS. Furthermore, despite lack of non-inferiority of TC, the present analysis demonstrated a small DFS deficit of 1.31% for TC and narrows the difference between the two chemotherapy regimens by further reducing the 95% CI of the treatments effect on DFS and OS. The results of our study are in accordance with a recent meta-analysis which demonstrated equivalent efficacy of TC to anthracycline-based regimens in terms of OS in the adjuvant setting, with a 43% probability of TC being the best, among a variety of chemotherapy agents [14].

With the exception of febrile neutropenia and the development of immune-related reactions, the overall pooled-toxicity profile favored TC. In general, taxanes are associated with toxicities such as myelosuppression and neuropathy [13]. In our pooled-analysis patients who received TaxAC experienced higher possibility of grade 3–4 neurotoxicity. Apart from neurotoxicity, cardiotoxicity prevailed with anthracycline use, while of note were the development of thrombocytopenia fatigue, nausea, vomiting, mucositis, pain and hand/foot syndrome in the TaxAC arm. Febrile neutropenia was more common in the TC group, but we need the data of the percentage of patients who received prophylactic G-CSF in each group before coming to any conclusion.

The cardiac toxicity of anthracyclines is well established and typically manifests as congestive heart failure, may develop up to 10 or 15 years after completion of treatment [5, 8, 20, 23]. Some populations are more susceptible to anthracycline toxicity [8], among them are women with left-sided tumors due to the administration of adjuvant radiotherapy [5, 24] and there is no doubt that increased age is oftenly associated with decreased physiological reserves and increased likelihood of comorbidities such as cardiovascular disease [13, 25, 26]. Finally, the concurrent administration of anthracyclines and taxane has been associated with the highest risk of hospitalization during therapy for EBC [15], reflecting an additional burden to patients and increased costs.

Our analysis has several limitations. Although no heterogeneity was found between the trials, the anthracycline-based regimens were different. The ABC trials used a DFS endpoint that included only invasive disease, excluding ductal carcinoma *in situ* as a recurrence endpoint. Also, we observed a discord between the trials concerning the definition of the high-risk EBC population. Finally, our study’s inability to confirm non-inferiority of TC might stem from the particularly conservative threshold that we choose and further analysis might be needed to determine a more fitting boundary of inferiority.

Although our pooled analysis failed to show non-inferiority of the TC regimen compared to TaxAC, we demonstrated that the absolute difference in DFS is relatively limited (1.28%). Whether this difference in clinically meaningful has to be discussed with each individual patient taking into account the estimated risk of disease relapse, the acute and late toxicities of TaxAC and of course patient’s perspective and wishes.

## MATERIALS AND METHODS

### Criteria

We reviewed the published literature and the proceedings of major oncology meetings for studies that fulfill the following criteria: 1) randomized controlled trials comparing the non-inferiority of the TC regimen to the ‘standard of care’ TaxAC, 2) early stage breast cancer, 3) HER2-negative adenocarcinoma and 4) high risk of recurrence. High risk was heterogenous in the trials and could be defined by any of the following conditions; the presence of lymph node metastasis, or, in the absence of lymph node disease (i.e. N0) patients would have negative endocrine receptors (ER), or, tumor size would be > 2.0 cm, or pT ≥ 2, or, smaller tumors (i.e. T1c) in the context of grade 3 histology or high OncotypeDX score (≥ 25 for B-46-I/07132 and B-49, or ≥ 31 for USOR 06-090). Finally, the study population consisted of women with an Eastern Cooperative Oncology Group performance status of 0 or 1, or with adequate hematopoietic, hepatic, and renal functions and a left ventricular ejection fraction of at least 50%.

Three trials were identified: the WSG Plan B trial, the Hellenic trial conducted by HORG and the joined efficacy analysis of the ABC trials. The ABC trials consisted of three individual, sequentially conducted, open label, randomized phase III trials, composed from USOR 06-090, NSABP-USOR and USOR 06-0690; the ABC trials efficacy data were never analyzed individually before they were combined. The present analysis was concluded by pooling data from all the above “five” randomized controlled trials (RCTs).

### Outcomes

The objective of this study was to address whether TC is non-inferior to TaxAC in the adjuvant setting of patients with HER2-negative, breast cancer. The outcomes analyzed were DFS and OS; DFS was considered the primary outcome. Recurrence of the primary cancer (local or distant), diagnosis of a second primary, or death from any cause were considered as DFS events. Overall survival was defined from the time of randomization to death from any cause. We analyzed separately the effect of the treatment on DFS in patients with lymph node-negative disease. The combined effect of the treatment on DFS or OS was expressed as a pooled Hazard Ratio (HR) of the anthracycline-free TC arm over the TaxAC arm. Thus, a HR over 1 would favor patients receiving the TaxAC regimen, whereas a HR<1 favors the anthracycline-free treatment.

Adverse events (AE) from the included studies were pooled to study differences in the toxicity profile between the treatment regimens. The risk of any AE was expressed as odds ratio (OR) by approximating the number of patients experiencing the AE in the anthracycline-free TC treatment arm by the population of that arm over the number of patients experiencing the AE in the TaxAC treatment arm by the population of that arm. Thus, an OR over 1 indicated higher risk of toxicity for the TaxAC arm, whereas OR<1 indicated higher risk of an AE for the TC group.

### Data extraction

The following characteristics were extracted from each published report: author’s name, year of publication, study design, regimen details, sample size and allocated patients, tumor characteristics (grading, ER-status), lymph node infiltration, menopausal status, type of resection, median follow-up, AEs, HRs along with 95% CIs and survival percentages (OS and DFS) at each study’s designated time point (i.e. a 5, 4 and 3 year survival rate was designated in the Plan B, the ABC and the HORG trials respectively). Extraction was performed only for published information. Intention to treat data were used for the pooled analysis.

### Statistical analysis

A HR of 1.18 for TC versus TaxAC, which corresponded to the threshold defined in the ABC trials, was chosen to demonstrate inferiority, as it was the most conservative measure among the included studies. For non-inferiority to be established, the upper boundary of the 95% CI for the treatment effect on DFS of the TC over the TaxAC arm, should be equal or less to 1.18.

The Inverse-Variance (IV) statistical method was applied for calculation of the pooled HRs. Percentages for survival (DFS and OS), were pooled according to arcsine square root transformation (Freeman and Tukey, 1950). Between studies heterogeneity was evaluated with Cochran’s *Q* test; in case of statistically significant heterogeneity (*Q* test *P* < 0.1) the Random Effects (RE) model was reported; otherwise the Fixed Effects (FE) model was adopted to estimate the pooled ratios. Higgins I^2^-statistic was used to quantify the degree of of inconsistency in the results. Visual inspection of a Standard Error/Effect Size funnel plot of the primary outcome as well as estimation of Egger’s test [27], were employed to assess publication bias. Statistical significance was set at the two-sided 0.05 level. RevMan software V5.3 and MedCalc V16.4 were used for the completion of the pooled data analysis.

Database screening/study selection, data extraction and quality assessment were performed each time by two researchers (PN and NS respectively); all researchers worked independently and in a blinded manner. Any disagreement was resolved by a third author (ES). All authors had full access to all data in the study and take responsibility for the integrity of the data and accuracy of the data analysis.

## SUPPLEMENTARY MATERIALS FIGURES


