# Supplementary material for: Taxane & cyclophosphamide vs anthracycline & taxane-based chemotherapy as adjuvant treatment for breast cancer: a pooled analysis of randomized controlled trials by the Hellenic Academy of Oncology
Source: Oncotarget. 2019 Feb 5;10(11):1209–16. doi: 10.18632/oncotarget.26632 (PMC6383821; doi:10.18632/oncotarget.26632)
Supplement: Supplementary file 1 [file oncotarget-10-1209-s001.pdf]

Taxane & cyclophosphamide vs anthracycline & taxane-based chemotherapy as adjuvant treatment for breast cancer: a pooled analysis of randomized controlled trials by the Hellenic Academy of Oncology

SUPPLEMENTARY MATERIALS

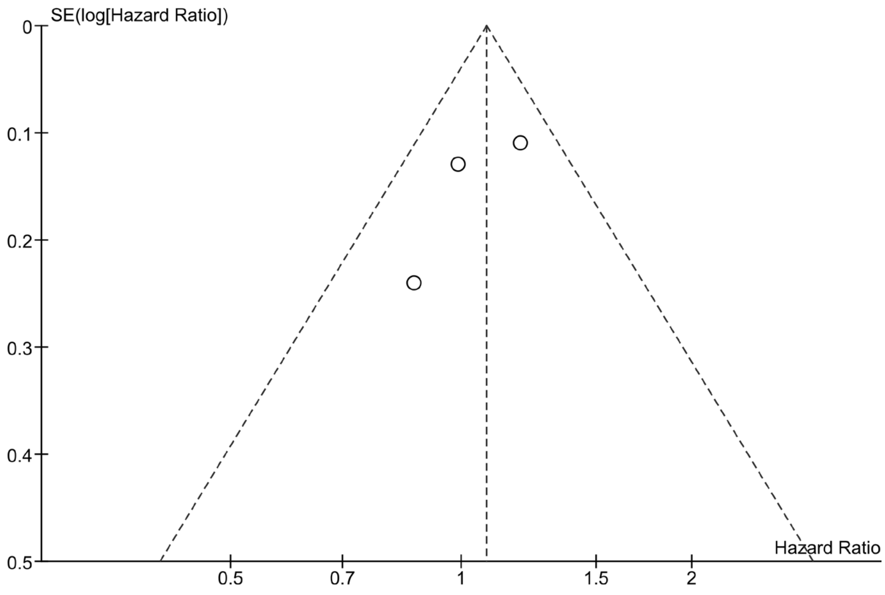

Supplementary Figure 1: Publication bias. SE= standard error.

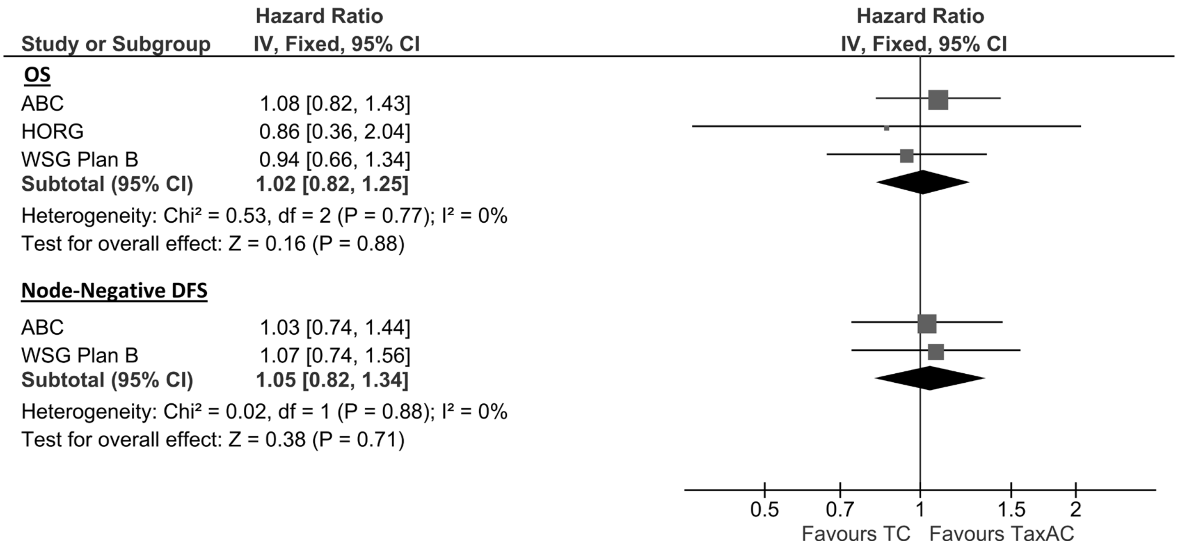

Supplementary Figure 2: Overall survival & node-negative DFS. OS = overall survival, DFS = disease free survival, CI = confidence interval, TC = taxane & cyclophosphamide, TaxAC = taxane & cyclophosphamide & anthracycline.
